# Rethinking immune checkpoint blockade: ‘Beyond the T cell’

**DOI:** 10.1136/jitc-2020-001460

**Published:** 2021-01-19

**Authors:** Xiuting Liu, Graham D Hogg, David G DeNardo

**Affiliations:** 1 Department of Medicine, Washington University School of Medicine in Saint Louis, Saint Louis, Missouri, USA; 2 Siteman Cancer Center, St. Louis, Mo, USA

**Keywords:** CTLA-4 Antigen, immunotherapy, immunity, innate, programmed cell death 1 receptor, tumor microenvironment

## Abstract

The clinical success of immune checkpoint inhibitors has highlighted the central role of the immune system in cancer control. Immune checkpoint inhibitors can reinvigorate anti-cancer immunity and are now the standard of care in a number of malignancies. However, research on immune checkpoint blockade has largely been framed with the central dogma that checkpoint therapies intrinsically target the T cell, triggering the tumoricidal potential of the adaptive immune system. Although T cells undoubtedly remain a critical piece of the story, mounting evidence, reviewed herein, indicates that much of the efficacy of checkpoint therapies may be attributable to the innate immune system. Emerging research suggests that T cell-directed checkpoint antibodies such as anti-programmed cell death protein-1 (PD-1) or programmed death-ligand-1 (PD-L1) can impact innate immunity by both direct and indirect pathways, which may ultimately shape clinical efficacy. However, the mechanisms and impacts of these activities have yet to be fully elucidated, and checkpoint therapies have potentially beneficial and detrimental effects on innate antitumor immunity. Further research into the role of innate subsets during checkpoint blockade may be critical for developing combination therapies to help overcome checkpoint resistance. The potential of checkpoint therapies to amplify innate antitumor immunity represents a promising new field that can be translated into innovative immunotherapies for patients fighting refractory malignancies.

## Introduction

Cancer research was transformed with the discovery that tumor-specific T cell dysfunction was reversible with immune checkpoint blockade.^1^ Antagonistic antibodies targeting cytotoxic T-lymphocyte-associated protein 4 (CTLA-4) and programmed cell death protein-1 (PD-1) or programmed death-ligand-1 (PD-L1) stimulate antitumor immunity and are now approved therapies in many cancer types, including metastatic melanoma and non-small cell lung cancer.^2^ These clinical successes highlight the immense potential for T cell-directed immunotherapy in cancer; however, we are just beginning to understand the full molecular activity of such agents. The remarkable achievements of these therapies in the clinic have elevated the T cell above all other immune lineages in the realm of antitumor immunity. Thus, the scope of research into immune checkpoint blockade may have been limited by ‘T cell centrism’. Growing evidence, reviewed below, highlights the emerging appreciation that innate immune cells mediate key aspects of checkpoint therapy biology. Despite numerous clinical successes, many patients do not respond to checkpoint therapies, and some cancer types are almost entirely resistant. An improved understanding of the mechanisms by which current checkpoint inhibitors function will enable clinicians to broaden the benefits of these treatments to greater numbers of patients.

### Keeping T cells in check

PD-1 and its ligands are central to regulating inflammation and peripheral tolerance. PD-1-null mice spontaneously develop a lupus-like syndrome due in part to uncontrolled proliferation of autoreactive T cells.^3^ PD-1 restrains T cell activity when engaged by its ligands, PD-L1 and PD-L2.^4^ PD-L1 expression is inducible in a variety of cell types, including adaptive and innate immune cells, mesenchymal cells, and cancer cells.^4^ In contrast, expression of PD-L2 is limited to antigen-presenting cells (APCs) and a smaller subset of tumor cell types.^4^ PD-1/PD-L1 signaling profoundly modulates T cell cytokine secretion, dampens T cell receptor (TCR) signaling, and shortens synapse engagement between T cells and APCs, resulting in impaired antitumor immunity.^5^ PD-1/PD-L1 blockade partially reverses these negative effects, augmenting T cell proliferation, tumor infiltration, and cytotoxicity.^4^

CTLA-4 is another crucial T cell coinhibitory receptor, which is upregulated in activated T cells and natively expressed by regulatory T cells (Tregs).^6^ In resting T cells, CTLA-4 is stored within cytosolic endosomes.^6^ After TCR engagement and costimulatory signaling via CD28, CTLA-4 molecules translocate to the cell surface, where they outcompete CD28 for its ligands, B7.1 and B7.2, expressed in APCs, restraining proliferation and activation of T cells.^6^ CTLA-4 has a non-redundant immunosuppressive role; CTLA-4-deficient mice die at 1 month of age as a result of a lethal lymphoproliferative disorder.^7^ In multiple models, CTLA-4 blockade results in T cell-mediated tumor rejection.^1^ These findings spurred clinical trials that demonstrated the efficacy of anti-CTLA-4 in multiple cancers as a single agent or in combination with anti-PD-1.^2^

#### The impact of checkpoint inhibitors on innate immune cells

**Figure 1:**
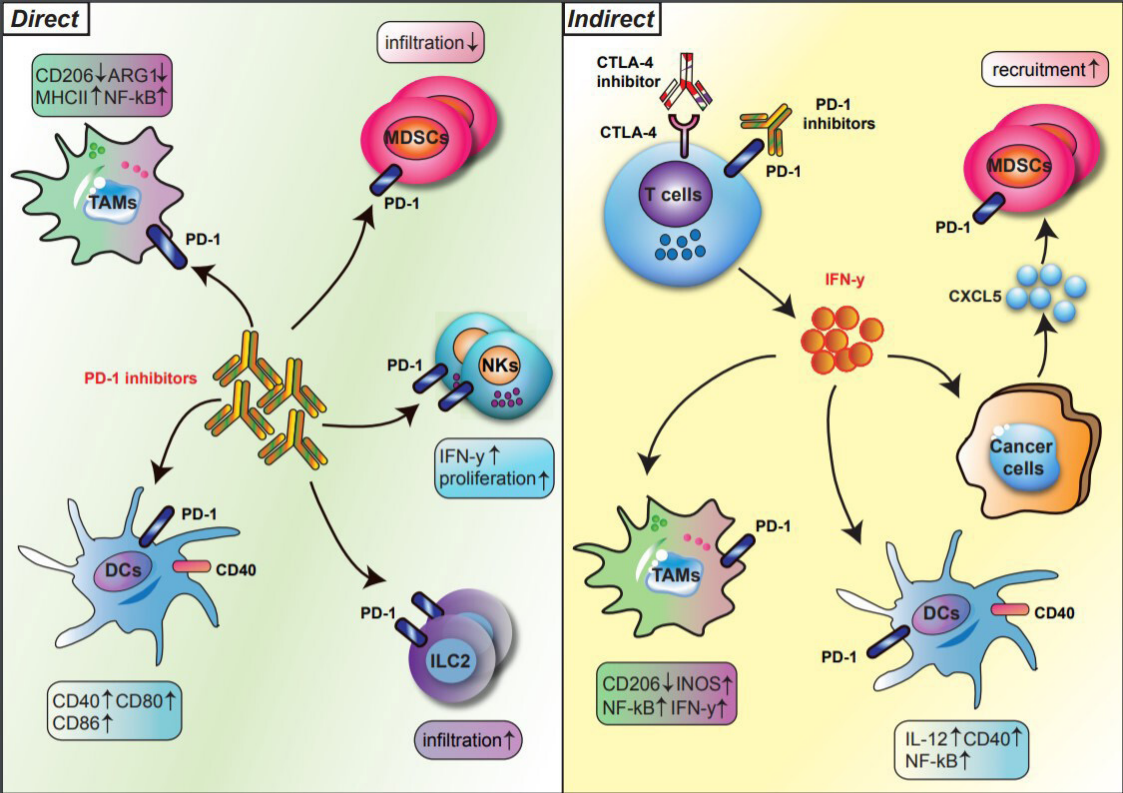
Direct and indirect regulation of innate immune subsets by PD-1 blockade. The regulation of innate immune cells by PD-1 blockade is divided into direct (left) and indirect (right) pathways. In the direct pathway, PD-1 blockade reshapes the phenotypes and functions of innate immune subsets, such as TAMs, DCs, MDSCs, NK cells, and ILC2s, expressing PD-1 (left). In the indirect pathway, T cells activated by anti-PD-1 secrete IFN-y, which in turn phenotypically polarizes myeloid cells within the TME (right). Bold arrows indicate interactions. DCs, dendritic cells; IFN-y, interferon gamma; ILCs, innate lymphoid cells; MDSCs, myeloid-derived suppressor cells: NK, natural killer cells; PD-1, programmed cell death protein 1; TAMs, tumor-associated macrophages; TEM, tumor microenvironment.

Over the last two decades, research on checkpoint inhibitors has focused on the T cell as the principal therapeutic target; however, recent studies have highlighted the significant effects of checkpoint inhibitors on innate immune cells. Checkpoint blockade has both a direct and an indirect impact on innate immune lineages (figure 1). In the indirect pathway, anti-PD-1/PD-L1 or anti-CTLA-4 reinvigorates T cell immunity, which, in turn, shapes or phenotypically polarizes innate immune cell responses within the tumor microenvironment (TME). In the direct pathway, innate immune cells are direct targets of immune checkpoint blockade because subtypes of myeloid cells and innate lymphocytes express PD-1 and/or PD-L1. This highly nuanced interplay of cell types after checkpoint therapy testifies to the importance of investigating how checkpoint biology affects innate immune populations.

### Tumor-associated macrophages and other myeloid cells in PD-1/PD-L1 checkpoint blockade

#### Indirect regulation

Macrophage function is orchestrated by activated T cells.^8^ T cell-associated cytokines such as interferon gamma (IFN-γ) stimulate macrophages to increase expression of major histocompatibility complex (MHC) molecules, costimulatory receptors, and the Th1-polarizing cytokine IL-12.^9^ Accordingly, checkpoint blockade-activated T cells dramatically alter phenotypes of tumor-associated macrophages (TAMs) and monocytes. Gubin and colleagues used single cell RNA-sequencing (scRNA-seq) and mass cytometry to assess transcriptional and functional changes in tumor-infiltrating myeloid cells after treatment with anti-PD-1.^10^ PD-1 blockade resulted in a reduction in CD206^+^ TAMs and an increase in inducible nitric oxide synthase^+^ (iNOS^+^) TAMs.^10^ This INOS^+^ TAM cluster was enriched for genes involved in IFN-γ signaling, high glycolytic activity, and NF-κB activity, suggestive of antitumor potential.^10^ Moreover, on IFN-γ neutralization, anti-PD-1-mediated repolarization of TAMs/monocytes was significantly diminished.^10^ These results demonstrate the potential of checkpoint-activated T cells to secrete factors, such as IFN-γ, that remodel the TME toward a tumor hostile environment, rich in iNOS^+^ TAMS, which are associated with improved outcomes in many tumor models. Thus, anti-PD-1 can be added to a growing list of therapies aimed at ‘repolarizing’ TAMs away from a tumor permissive phenotype.

#### Direct regulation

Myeloid cells display detectable PD-1 levels.^11^ PD-1 expressing TAMs have been shown to promote tumor progression in several cancers including gastric cancer,^12^ colorectal cancers,^13^ and lung cancer.^14^ The appearance of PD-1 in myeloid progenitors is an early event in tumor progression, as the receptor may be induced by inflammatory conditions.^15^ Indeed, bone marrow–derived macrophages (BMDMs) rapidly upregulate PD-1 after Toll-like receptor (TLR)−2 engagement^16^; correspondingly, PD-1 was upregulated by macrophages in a murine model of sepsis.^17^ The signaling downstream of PD-1 in macrophages is controversial and may be contextually dependent. PD-1-null BMDMs express more IL-6, and CCL2 (MCP-1) at 4-hour post TLR2 stimulation, suggesting an anti-inflammatory role of PD-1.^16^ In contrast, in vivo evidence found the opposite effect. Septic PD-1-null mice demonstrate decreased levels of peritoneal CCL2, tumor necrosis factor-alpha (TNF-α), and IL-1β, an observation that was abrogated on depletion of peritoneal macrophages.^17^ These in vivo data suggest that PD-1 expression on macrophages augments systemic inflammation. Despite the lack of a clear signaling pathway downstream of PD-1 in macrophages, there is a clear link between TAM expressed PD-1 and cancer-associated inflammation. In cancer models, synergistic antitumor effects were found with the combination of PD-1/PD-L1 blockade and neutralization of either IL-6^18^ or IL-1β,^19^ indicating that targeting the inflammatory TME could amplify PD-1 blockade efficacy. This effect likely involves macrophage expressed PD-1, as the authors found that PD-1 agonism suppressed production of IL-6 by PD-1-bearing TAMs, whereas anti-PD-L1 enhanced IL-6 production.^18^ Thus, anti-inflammatory therapies may be necessary for maximizing the benefit of checkpoint blockade. These data suggest a complex role of myeloid intrinsic PD-1 signaling and highlight the need to delineate the mechanistic differences between PD-1 blockade and PD-1 deletion.

New model systems have permitted the investigation of the myeloid-specific effects of checkpoint blockade. Strauss *et al* generated a mouse model in which PD-1 was selectively deleted in myeloid cells.^15^ The authors employed these mice to dissect the relative contribution of myeloid versus T cell PD-1 signaling in colon cancer.^11^ Interestingly, myeloid-specific PD-1 deletion was as effective at limiting tumor growth as global PD-1 deletion, and more effective than selective ablation of PD-1 in T cells.^15^ One caveat to these studies is that genetic approaches to interrupt PD-1/PDL-1 signaling may not accurately model therapeutic antagonist therapies. However, the authors treated Recombination Activating Gene-2-null mice lacking T and B cells with anti-PD-1 and still observed a significant reduction in tumor growth,^15^ again emphasizing the critical importance of the innate immune system for checkpoint blockade.

**Figure 2:**
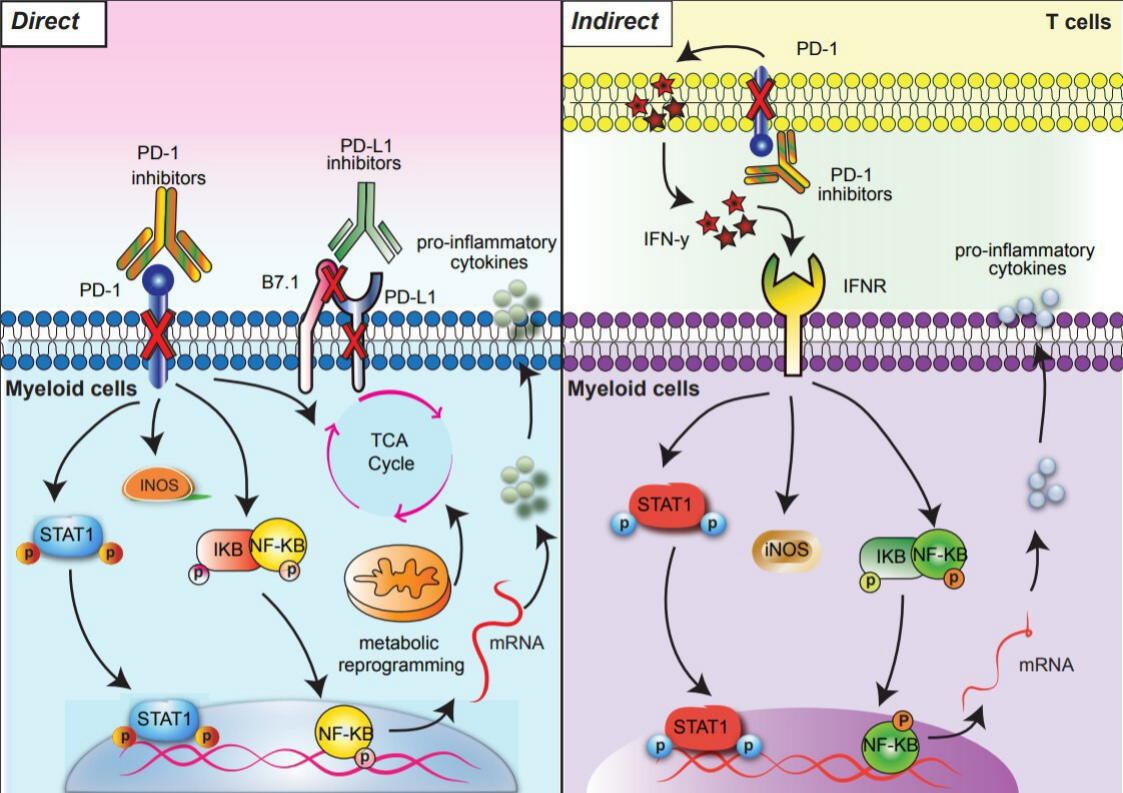
Direct and indirect signaling pathways downstream of PD-1 blockade in myeloid cells. PD-1 blockade results in direct (left) and indirect (right) signaling outcomes. Direct PD-1 blockade in PD-1 expressing myeloid cells activates NF-κB and pSTAT1 signaling pathways and reprograms glycometabolism (left). In the indirect pathway, anti-PD-1 activated T cells secrete IFN-y which triggers NF-κB and pSTAT1 signaling pathways in myeloid cells (right). Arrows indicate downstream outcomes of PD-1 blockade. IFN-γ, interferon gamma; PD-1, programmed cell death protein 1.

PD-1 engagement on myeloid cells affects infiltration, differentiation, effector function, and cellular metabolism. Some of these pathways and outcomes are highlighted in figure 2. PD-1 engagement shifts activated human monocyte metabolism toward oxidative phosphorylation. PD-1/PD-L1 blockade is able to rescue glycolysis, which is correlated with enhanced antibody-dependent phagocytosis.^20^ PD-1-deficient myeloid cells exhibit altered development from common myeloid progenitors, with diminished accumulation of granulocyte/macrophage progenitors in the bone marrow and increased expansion of Ly6C^+^ monocytes and dendritic cells (DCs) within the tumor.^15^ These data suggest that PD-1 signaling in myeloid progenitors may direct myelopoiesis toward the granulocytic lineage, resulting in greater numbers of immunosuppressive granulocytic-MDSCs. These findings suggest that checkpoint therapies may benefit from drug combinations that limit tumor infiltration by myeloid subsets. Regarding effector function, PD-1 expressing TAMs demonstrate high levels of CD206, arginase 1 (ARG1), and IL-10, which dampen antitumor immune responses.^13^ In contrast, PD-1 deficiency in TAMs shifts their phenotype toward an antitumor profile, with higher levels of TNFα, iNOS, and MHCII.^21^ In multiple cancer models, TAM infiltration is skewed toward CD206^+^, ARG1^high^ macrophages^22^; however, anti-PD-1 therapy reverses this trend, increasing the expression of iNOS, TNF-α, and IL-6, which may augment antitumor immunity.^14^ These findings corroborate the scRNA-seq results of Gubin *et al* and strikingly highlight that at a transcriptomic level, checkpoint therapy has a concomitant, if not greater, impact on TAM phenotype than on T cell phenotype. Together, these data suggest that PD-1 blockade reprograms TAMs toward an antitumor phenotype.

#### Myeloid-specific effects of PD-L1 blockade

Canonically, PD-L1 interacts with its receptor PD-1 on tumor-specific T cells and limits their antitumor activity.^23^ Anti-PD-L1 therapy blocks this interaction, thereby reinvigorating T cell proliferation and effector functions such as IFN-γ secretion.^24^ However, like anti-PD-1, PD-L1 blockade can also directly and indirectly modulate myeloid cell function. Anti-PD-L1 has been shown to indirectly repolarize TAMs toward a proinflammatory phenotype, in a T cell-dependent, IFN-γ-mediated process.^25^ These anti-PD-L1-treated TAMs exhibit decreased expression of ARG1 and enhanced iNOS, MHCII, and CD40 expression, indicative of an antitumor phenotype.^25^ In the direct pathway, TAMs can engage with activated T cells expressing PD-L1. T cell PD-L1 binds TAM-expressed PD-1 and induces a tolerogenic phenotype.^26^ These findings indicate that anti-PD-L1 may disrupt multiple axes of PD-1 engagement to restore the antitumor potential of TAMs.

The bulk of research on anti-PD-L1 therapy emphasizes the disrupted interaction between tumor-expressed PD-L1 and T cell-expressed PD-1. However, PD-L1 is widely inducible on immune subsets, tumor cells, and even endothelial cells, in an IFN-γ-dependent process.^27^ Thus, both tumor and host PD-L1 are intimately involved in checkpoint blockade.^28^ In some malignancies such as colon cancer, tumor immune infiltrates express PD-L1 at significantly greater levels than tumor cells.^29^ Thus, it is essential to study the role of tumor extrinsic PD-L1. Indeed, PD-L1 expressing lymph node resident APCs can inhibit T cell activation and prevent recruitment of primed T cells to the TME.^30^ PD-L1^+^ neutrophils have been shown to impair T cell immunity in hepatocellular carcinoma.^31^ In some models, PD-L1 expression by cancer cells is dispensable for anti-PD-L1 efficacy.^28 30 32^ PD-L1 blockade efficacy was retained in a PD-L1-deficient model of colon adenocarcinoma.^30^ However, efficacy was lost if the bone marrow from PD-L1-null mice was transplanted into tumor-bearing mice, indicating that a hematopoietic cell was responsible for the response.^30^ The efficacy was again lost after depleting CD11b^+^ PD-L1^+^ cells, presumably due to an absence of PD-L1-expressing APCs.^30^ These data support the essential role of PD-L1^+^ myeloid cells in anti-PD-L1 therapy. Other authors have disputed the dispensable nature of tumor expressed PD-L1 and have demonstrated that both tumor and APC expressed PD-L1 are involved in critical but distinct aspects of PD-1/PD-L1 blockade.^28 32^ These findings have broad implications for the use of PD-L1 as a biomarker for checkpoint efficacy. Patients may benefit from separate quantification of tumor and tumor immune infiltrate PD-L1 expression; and differing cancer types may have divergent biology herein.

The portrait of myeloid PD-L1 engagement is further complicated by in vitro evidence that PD-L1 can have intrinsic signaling of its own. When engaged, PD-L1 can induce proliferation, costimulatory molecule expression, cytokine production, and mTOR signaling in macrophages.^33^ It is noteworthy that TAMs can express both PD-L1 and its ligands PD-1 and B7.1 (CD80), which have sufficient affinity to interact in cis.^34^ The evidence presented above suggests that PD-L1 inhibitors may have intrinsic effects on myeloid cells by either preventing direct engagement in trans or disrupting cis interactions. Further research is needed to elucidate the primary mode of myeloid specific anti-PD-L1 efficacy. Potential modalities include inhibition of PD-1 engagement on myeloid cells, disruption of myeloid PD-L1 engagement with T cell PD-1 or inhibition of direct signaling through myeloid PD-L1. Regardless of the primary modality, it is increasingly appreciated that blocking PD-L1 may have a very different outcome compared with blocking PD-1. Indeed, in a model of pancreatic cancer, the combination of PD-1 and PD-L1 confer a synergistic benefit over either therapy alone.^35^ Based on the essential role of PD-1/PD-L1 in myeloid cells, it is likely that the success of anti-PD-L1 and anti-PD-1 combined therapy relies both on the disruption of immunosuppressive TAM-T cell crosstalk and on directly reshaping TAM phenotype toward an antitumor profile.

### Linking PD-1/PD-L1 blockade and DC function

#### Indirect regulation

The efficacy of PD-1 blockade may depend on the indirect activation of tumor-infiltrating DCs. Anti-PD-1-activated T cells secrete IFN-γ, which in turn sets in motion a dramatic transcriptomic shift in DC phenotype, as they express antigen presentation machinery, upregulating IL-12, CCR7, MHCII, CD80, CD40, TLR-2, and TLR-4.^36 37^ CCR7 enables DC trafficking to tumor draining lymph nodes, where DCs prime CD8 and CD4 T cell responses via class I and II MHC and the costimulatory molecules CD80/CD86.^36 37^ Signaling through CD40 and TLRs further augments IL-12 production, which activates CD8 T cells and polarizes primed CD4 T cells toward the Th1 subset, which in turn secrete additional IFN-γ in a feed forward loop.^36–38^ In summary, IFN-γ-stimulated DCs are highly efficient APCs, specialized for priming in vivo T cell responses. Recent studies have demonstrated that DCs are also necessary for anti-PD-1 efficacy. In a fibrosarcoma model, anti-PD-1-mediated tumor regression was lost when DCs were depleted or when IL-12 was neutralized.^38^ Additionally, tumor-bearing mice with DCs expressing a conditional mutation in the IFN-γ receptor demonstrate profoundly depressed IL-12 production, and lose checkpoint responsiveness.^38^ Interestingly, these IL-12^+^ DCs are enriched for non-canonical NF-κB signaling pathway components, such as CD40, Nfkb2, and Relb.^38^ Indeed, inactivation of the non-canonical NF-κB pathway also abrogates checkpoint efficacy.^38^ These observations have motivated researchers to combine CD40 agonists with PD-1/PD-L1 blockade due to potential synergy.^38^ Improving T cell-DC crosstalk via non-canonical NF-κB signaling offers the compelling possibility of converting the TME from immunologically cold to checkpoint responsive.

#### Direct regulation

Although indirect regulation of DCs by anti-PD-1 is well established, evidence of direct regulation is still emerging. Patients with hepatocellular carcinoma^39^ and ovarian cancer^40^ have increased numbers of PD1-bearing DCs, suggesting that the expression of PD-1 by DCs may be context-dependent. Emerging evidence indicates that PD-1 signaling in DCs may inhibit survival^41^ and decrease secretion of IL-12 and TNF-α, suppressing the antitumor potential of CD8^+^ T cells.^42^ PD-1 signaling in DCs also engages the canonical NF-κB pathway and suppresses antigen presentation machinery by blocking surface expression of MHCI.^40 43^ In vivo models of hepatocellular carcinoma support an immunosuppressive role for PD-1 in DCs. Specific ablation of PD-1 on intratumoral DCs resulted in enhanced priming of tumor-specific CD8 T cells, which exhibited increased expression of the cytolytic molecules perforin and granzyme-B.^39^ Additionally, PD-1 inhibition increases DC expression of the costimulatory molecules, CD40, CD80, and CD86,^43^ which may be due to increased MAPK signaling.^41^ The above studies demonstrate that PD-1 regulates DC function both directly and indirectly within the inflammatory TME. In some models, anti-PD-1 efficacy indirectly requires components of the non-canonical NF-κB pathway, and in others, anti-PD-1 directly drives upregulation of components such as CD40. Thus, multiple lines of evidence suggest the combination of CD40 agonists and anti-PD-1 as a means of improving antitumor immunity and overcoming checkpoint resistance.

### DC-specific effects of PD-L1 blockade

Anti-PD-L1 therapy is now employed to treat numerous solid tumor types; however, the mechanism of action still remains debated. One clue toward this mechanism is the recent observation that expression of PD-L1 by intratumoral immune infiltrate is a better correlate to anti-PD-L1 clinical response than tumor expressed PD-L1.^44^^44^ Interestingly, a DC-specific transcriptomic signature, calculated from the expression levels of XCR1, BATF3, IRF8, and Flt3, stratified anti-PD-L1-treated renal cell carcinoma and lung cancer patients into long-term and short-term survivors, suggesting that DC-expressed PD-L1 may be a primary target of successful anti-PD-L1 therapy.^45^ PD-L1 is widely expressed in DC subsets, with increased expression in the setting of inflammation and cancer.^45 46^ DC-expressed PD-L1 acts as a homeostatic control of autoimmunity and directly restrains highly active T cell responses.^47^ In two recent publications, researchers sought to study the contribution of DCs to anti-PD-L1 therapy by conditionally deleting PD-L1.^48 49^ In both cases, selective ablation of PD-L1 on DCs restricted tumor growth as effectively as systemic PD-L1 knockout mice, indicating that DCs are likely critical to the reinvigorated T cell response after anti-PD-L1 immunotherapy.^48 49^ At early timepoints, DC-specific PD-L1 deletion led to enhanced tumor infiltration of effector CD8^+^ T cells, but not proliferation, indicating there may be increased T cell recruitment.^48^ These findings highlight that checkpoint-responsive patients may be best identified by measuring DC expression of PD-L1 rather than tumor expression.

Aside from the classical PD-1/PD-L1 axis, APC-expressed PD-L1 can also bind the costimulatory molecule B7.1 (CD80) in cis with higher affinity than the canonical T cell costimulatory receptor CD28.^34^ Consequently, T cell immunity may be suppressed by both the PD-L1−B7.1 and the PD-L1−PD1 axes, either by directly dampening TCR signaling or by restricting a necessary costimulatory signal.^50^ In two recent publications, researchers reached differing conclusions as to whether blocking PD-L1’s cis interaction enhances CD28 signaling.^45 48^ Disrupting the PD-L1−B7.1 interaction may in fact accelerate tumor growth by permitting PD-L1/PD-1 engagement; however, this observation was accompanied by a significant increase in intratumoral T cell numbers, indicating that enhanced priming via CD28-B7.1 may have also occurred.^48^ Further research is needed to clarify the role of cis PD-L1−B7.1 binding on DCs, and whether DC-specific PD-L1 engages PD-1 expressed by other myeloid subsets such as TAMs. DCs are uniquely specialized APCs that efficiently prime naive T cell responses.^51^ The evidence above demonstrates that DCs are critical for anti-PD-L1 efficacy. Thus, PD-L1 blockade may offer a targeted approach to improve DC-mediated priming of antitumor T cell responses, by both releasing B7.1 and disinhibiting downstream TCR activation.

### The complex role of MDSCs in PD-1/PD-L1 blockade

MDSCs are a heterogeneous group of relatively immature myeloid cells, which can play an immunosuppressive role in multiple cancers.^52 53^ The effects of PD-1/PD-L1 blockade on MDSCs are multifaceted and represent a double-edged sword because they may simultaneously induce antitumor immunity and promote tolerance.

#### Indirect regulation

While anti-PD-1/PD-L1 therapies have shown potential to induce antitumor myeloid activity, they can also have complex protumorigenic outcomes, such as recruiting immunosuppressive MDSCs.^54^ Circulating MDSCs are associated with poor survival in patients with melanoma who have received checkpoint therapy.^53^ The frequency of tumor-infiltrating MDSCs is significantly elevated in metastatic melanoma biopsies obtained from patients receiving anti-PD-1 therapy.^55^ This recruitment may be driven by anti-PD-1-activated T cells secreting IFN-γ, which partially triggers the tumor-intrinsic NLRP3 inflammasome.^54^ NLRP3 activity drives CXCL5-mediated recruitment of granulocytic MDSCs.^54^ Thus, anti-PD-1 therapy can lead to anti-PD-1 resistance, in an apparent negative feedback loop. In addition to IFN-γ, checkpoint-activated CD8^+^ T cells secrete more TNF-α, both of which lead to tumor production of CSF1. CSF1, in turn, induces the differentiation and survival of protumorigenic TAMs and MDSCs, thus magnifying checkpoint resistance.^56^ Based on these data, one clear strategy to improve checkpoint efficacy is to limit the infiltration of MDSCs. Indeed, in models of pancreatic cancer, colon cancer, and breast cancer, blockade of CXCR2 suppresses the recruitment of MDSCs, triggering antitumor immunity, increasing T cell numbers, and sensitizing tumors to anti-PD-1 therapy.^57^ The above studies clearly demonstrate that PD-1/PD-L1 checkpoint blockade may indirectly drive checkpoint resistance by expanding and recruiting MDSCs. Treatment of multiple solid tumors may benefit from combining checkpoint therapy with targeted strategies to limit MDSC chemotaxis.

#### Direct regulation

MDSCs may express both PD-1 and PD-L1, which can lead to a reversal of MDSC-related immunosuppression when targeted by checkpoint blockade.^58–60^ As with other myeloid lineages, MDSC expression of PD-1 and PD-L1 is inducible in inflammatory settings.^58^ Activated T cells may promote MDSC expression of PD-L1 through the IFN-γ−IFNGR1−STAT1−IRF1 axis.^59^ MDSCs with high expression of PD-1/PD-L1 demonstrate high rates of proliferation, leading to their robust expansion in the TME of many cancers.^58^ The joint expression of PD-1 and PD-L1 by MDSCs suggests that both anti-PD-1 and anti-PD-L1 should be evaluated for their MDSC remodeling potential. Indeed, in a model of multiple myeloma, joint therapy with anti-PD-1 and anti-PD-L1 prevented MDSC-mediated cancer promotion to a greater extent than either alone.^60^ In contrast to cancers such as melanoma, head and neck squamous cell carcinoma (HNSCC) demonstrates reduced granulocytic MDSC infiltration post-PD-1/PD-L1 blockade.^61^ It is unclear whether this model involves different mechanisms of MDSC recruitment or whether the direct blockade of PD-1 is inhibiting MDSC proliferation. Antagonism of PD-L1 in MDSCs has been shown to reduce the immunosuppressive polarization of T cells. In coculture experiments, treatment of MDSCs with anti-PD-L1 led to increased rates of T cell proliferation and IFN-γ production, which may be an outcome of reduced IL6 and IL10 production.^62 63^ The studies discussed above highlight the nuanced and complex effects of checkpoint therapy on MDSCs. PD-1/PD-L1 blockade can improve outcomes via a direct reversal of MDSC-related immunosuppression, while synchronously driving checkpoint resistance through MDSC recruitment.

### Innate lymphocytes and PD-1/PD-L1 blockade

Recent studies have identified innate lymphocytes as novel targets of checkpoint inhibitors.^64 65^ Innate lymphocytes are divided into two large branches: natural killer (NK) cells and innate lymphoid cells (ILCs). ILCs are further divided into ILC1s, ILC2s, ILC3s, and lymphoid tissue-inducer (LTi) cells.^66^ NK cells functionally mirror CD8^+^ T cells because they exhibit antitumor cytotoxic activity. In comparison, ILC subsets are tissue-resident populations with analogous roles to CD4^+^ T helper (Th) cell subsets. ILC1s produce IFN-γ to control intracellular pathogens, ILC2s produce IL-4, IL-5, and IL-13 to target parasites, and ILC3s secrete IL-17 and IL-22 to defend against extracellular bacteria and fungi.^67^ LTi cells are critical for the formation of secondary lymphoid tissues.^67^

#### NK cells

Solid tumors, such as renal cell carcinoma, can be heavily infiltrated by NK cells.^66^ These innate lymphocytes are regulated by constitutively expressed activating and inhibitory receptors, which recognize stress-induced ligands and various conserved motifs on class I and non-canonical MHC molecules.^68^ There are conflicting reports as to whether NK cells express significant levels of PD-1. Some groups suggest that neither mouse nor human NK cells express PD-1;^69^ however, other groups have reported PD-1 expressing NK cells in several cancer types.^70^ It is likely that PD-1 is expressed by NK cells in certain inflammatory conditions. Tumor-educated NK cells upregulate PD-1, which, when engaged by PD-L1, dampens NK cell-mediated antitumor immunity.^71^ Emerging evidence suggests that PD-1/PD-L1 blockade increases both NK cell recruitment and cytotoxicity against multiple myeloma cells.^64^ Additionally, anti-PD-1 therapy is capable of triggering NK cell activation and production of IFN-γ.^72^ To further complicate matters, NK cells may express PD-L1, which induces enhanced antitumor functionality when agonized.^73^ In vivo studies suggest that the use of anti-PD-L1 not only blocks negative PD-1 engagement on NK cells, but also activates PD-L1^+^ NK cells, leading to enhanced tumor rejection.^73^ Although NK cells likely represent an additional target of PD-1/PD-L1 blockade, additional research is needed to determine whether NK cell-specific PD-1/PD-L1 blockade is clinically relevant in solid tumors, and whether a physiological role exists for PD-L1 signaling in these innate immune cells.

#### Innate lymphoid cells

Checkpoint inhibitors are also capable of reshaping ILC responses in pathological conditions such as cancer.^74^ While high PD-1 expression on ILC progenitors is lost on differentiation, PD-1 levels may be upregulated in response to tissue-specific cues. PD-1 is upregulated in tissue-resident ILC2s in the context of lung inflammation.^75^ The depletion of PD1^high^ effector ILC2s reduces inflammation during influenza and allergen exposure.^75^ Expression of PD1 on ILCs is also relevant for antitumor immunity. Tumor-infiltrating ILC2s (TILC2s) are predictive of long-term survival in patients with pancreatic cancer.^76^ TILC2s express significant levels of PD-1, and TILC2 density increases post-anti-PD-1 therapy.^76^ In an orthotopic model of pancreatic cancer, TILC2s adoptively transferred into ILC2-deficient hosts were partially responsible for the reduction in tumor burden post-anti-PD-1 therapy.^76^ In addition to ILC2s, ILC3s are also affected by checkpoint therapies. ILC3s express PD-1 in primary and metastatic tumors,^65^ and the PD-1/PD-L1 axis in ILC3s regulates cytokines secretion and immune tolerance.^77^ Although still in its early stages, research into the role of ILCs during checkpoint therapy represents a promising new field for cancer immunotherapy.

### The impact of CTLA-4 blockade on innate immune cells

Although CTLA-4 expression is largely restricted to T cell lineages and some cancers, there are numerous indirect effects of anti-CTLA-4 therapy on innate immune subsets. Anti-CTLA-4 blockade reduces the numbers of tumor-infiltrating MDSCs and protumorigenic TAMs in a spontaneous model of HNSCC.^78^ In addition, patients with advanced melanoma treated with anti-CTLA-4 exhibit decreased intratumoral MDSC numbers with a reversal in their tolerogenic profiles.^79^ As mentioned previously, Gubin *et al* demonstrated the ability of checkpoint blockade, including anti-CTLA-4 therapy, to reshape the myeloid compartment.^10^ Anti-CTLA-4 therapy indirectly polarizes TAMs, in an IFN-γ-dependent process, toward an antitumor phenotype characterized by the increased expression of NF-κB-related genes.^10^ Of note, a recent publication demonstrated that the efficacy of human anti-CTLA-4 was partially attributable to the Fc portion of the antibody and it’s affinity to Fcγ-receptors such as CD32a, expressed by multiple innate subsets.^80^ Treatment with anti-CTLA-4 resulted in depletion of CTLA-4 expressing Tregs, highlighting an additional role of innate subsets responsible for antibody-dependent-cellular cytotoxicity during checkpoint blockade.^80^ However, these effects may be highly dependent on the IgG class of the CTLA-4 targeting antibody. There is more limited evidence for the direct engagement of CTLA-4 on innate immune lineages. Subsets of tumor-infiltrating NK cells express CTLA-4 and CD28, and CTLA-4 blockade was found to inhibit IFN-γ release by NK cells on coculture with mature DCs.^81^ In summary, like anti-PD1/PD-L1 therapies, anti-CTLA-4 therapy is capable of inducing global shifts in tumor-infiltrating innate immune subsets. Reports of the expression of CTLA-4 and CD28 in tumor-infiltrating NK cells suggest that further efforts are needed to assess whether the NK cell-specific effects of anti-CTLA-4 blockade have been misattributed to T cells.

### Emerging checkpoints inhibitors and their impact on innate immune cells

The clinical success of checkpoint therapies targeting PD-1/PD-L1 and CTLA-4 spurred intense interest in identifying additional coinhibitory and costimulatory receptors as potential therapeutic targets. After PD-1 and CTLA-4, lymphocyte-activation gene 3 (LAG3) was the third inhibitory receptor to be clinically targeted.^82^ In lymphoid lineages, LAG3 is highly expressed in activated T cells, Tregs, and NK cells.^82^ In myeloid subsets, LAG3 is found in plasmacytoid DCs (pDCs), which are critical for mounting antiviral responses,^83^ and in TAMs in some cancer-specific conditions, such as B-cell lymphoma.^84^ Interestingly, LAG3-null mice have increased numbers of macrophages, granulocytes, and pDCs; thus, the receptor may play a role in regulating hematopoiesis.^83^ A population of Tregs coexpressing LAG3 and T-cell-immunoglobulin-and-mucin-domain-containing-molecule-3 (TIM3) suppress the proinflammatory activation of macrophages,^85^ suggesting that the blockade of LAG3 could synergize with the indirect effects of anti-PD-1/PD-L1 and/or anti-CTLA-4 therapies, which shift the TAM phenotype toward an antitumor state. Despite the known immunosuppressive role of LAG3 in T cells, research into the regulation of myeloid subsets by LAG3 is still limited.

Like LAG3, T-cell-immunoreceptor-with-Ig-and-ITIM-domains (TIGIT) is a coinhibitory receptor primarily expressed in T cells and NK cells. TIGIT interacts with a variety of nectin-like molecules expressed in APCs and various cancers, leading to impaired T and NK cell proliferation and IFN-γ production.^86^ Interestingly, MDSC-mediated suppression of NK cells may be partially TIGIT-dependent as an anti-TIGIT antibody was able to reverse NK cell dysfunction in MDSC coculture experiments.^87^ The mechanism underlying this finding may involve decreased Arg1 expression in MDSCs, as has been reported in the context of T cells.^88^ As with PD-1-null mice, TIGIT-null mice have reduced tumor growth in various cancer models,^89^ and NK cells isolated from these mice demonstrate enhanced IFN-y secretion when activated by target cells.^90^ Further research is needed to evaluate the indirect effects of anti-TIGIT blockade on myeloid subsets.

Unlike TIGIT and LAG3, TIM3 is broadly expressed and directly regulates both innate and adaptive immune subsets.^91^ Specifically, TIM3 is found in T cells, NK cells, macrophages, TAMs, DCs, and mast cells.^91^ TIM3 agonism reduces inflammatory signaling in both adaptive and innate cell types, and as such, is an attractive target for future immunotherapies.^92^ In T cells, TIM3 blockade increases Th1 proliferation and IFN-γ production.^93^ Recent findings highlight that NK cell cytotoxicity is also attenuated by TIM3 expression, which may play a role in maternal-fetal tolerance.^91^ In macrophages, TIM3 signaling dampens inflammation and limits phagocytic capacity.^91^ Additionally, blockade of TIM3 reduces MDSC recruitment and slows tumor growth in a model of HNSCC.^94^ Within the DC lineage, TIM3 is primarily expressed by CD8^+^/CD103^+^ DCs, which are specialized for priming CD8^+^ T cell responses through cross-presentation.^91^ The outcome of TIM3 signaling in DCs is varied and can have both immunosuppressive and protumorigenic effects. Treating a DC cell line with lipopolysaccharide and the TIM3 ligand galectin-9 significantly increased TNF-α production.^95^ However, TIM3 has also been shown to dampen inflammation by interfering with the engagement of DNA-specific TLRs.^96^ TIM3 blockade improves antitumor DC-T cell crosstalk by enhancing cross-presentation by CD103^+^ DCs,^91^ and increasing DC expression of CXCL9, a T cell chemoattractant.^97^ Interestingly, in a fibrosarcoma model, DCs were required for anti-TIM3-enhanced chemotherapy efficacy. These results suggest that TIM3 may inhibit DC activation, antigen presentation, and DC crosstalk in the setting of chemotherapy-induced immunogenic cell death.^96^ Taken together, TIM3’s wide expression in innate and adaptive subsets, and its demonstrated role in the crosstalk between the two, highlight TIM3 as a promising immunotherapeutic target. The emergence of novel coinhibitory receptors will necessitate careful evaluation of which targets synergize with existing checkpoint therapies and broadly enhance antitumor immunity in both innate and adaptive compartments.

## Conclusions

The studies highlighted in this review paint a complex picture of the relationship between checkpoint blockade and innate immunity. Myeloid cells and innate lymphocytes contribute to both checkpoint efficacy and resistance, through both direct and indirect mechanisms. The challenge for researchers and clinicians is to balance the varied, and sometimes opposing, effects of checkpoint therapies, so that they may intelligently employ and combine immunotherapies in the fight against cancer. Shifting the focus of checkpoint blockade from ‘T cell centrism’ to a more holistic view of the complex and interconnected TME may reveal new opportunities to broaden the benefits of checkpoint blockade to the many patients in need of prognosis-altering therapies.

## References

[1] Leach DR, Krummel MF, Allison JP Enhancement of antitumor immunity by CTLA-4 blockade. Science 1996;271:1734–6. 10.1126/science.271.5256.1734 8596936

[2] Wilky BA Immune checkpoint inhibitors: the linchpins of modern immunotherapy. Immunol Rev 2019;290:6–23. 10.1111/imr.12766 31355494

[3] Nishimura H, Nose M, Hiai H, et al Development of lupus-like autoimmune diseases by disruption of the PD-1 gene encoding an ITIM motif-carrying immunoreceptor. Immunity 1999;11:141–51. 10.1016/S1074-7613(00)80089-8 10485649

[4] Henick BS, Herbst RS, Goldberg SB The PD-1 pathway as a therapeutic target to overcome immune escape mechanisms in cancer. Expert Opin Ther Targets 2014;11:1–14. 10.1517/14728222.2014.955794 25331677

[5] Baumeister SH, Freeman GJ, Dranoff G, et al Coinhibitory pathways in immunotherapy for cancer. Annu Rev Immunol 2016;34:539–73. 10.1146/annurev-immunol-032414-112049 26927206

[6] Krummel MF, Allison JP Ctla-4 engagement inhibits IL-2 accumulation and cell cycle progression upon activation of resting T cells. J Exp Med 1996;183:2533–40. 10.1084/jem.183.6.2533 8676074 PMC2192613

[7] Tivol EA, Borriello F, Schweitzer AN, et al Loss of CTLA-4 leads to massive lymphoproliferation and fatal multiorgan tissue destruction, revealing a critical negative regulatory role of CTLA-4. Immunity 1995;3:541–7. 10.1016/1074-7613(95)90125-6 7584144

[8] Mark Doherty T, Doherty TM T-Cell regulation of macrophage function. Curr Opin Immunol 1995;7:400–4. 10.1016/0952-7915(95)80117-0 7546407

[9] Hsieh C, Macatonia S, Tripp C, et al Development of Th1 CD4+ T cells through IL-12 produced by Listeria-induced macrophages. Science 1993;260:547–9. 10.1126/science.8097338 8097338

[10] Gubin MM, Esaulova E, Ward JP, et al High-Dimensional analysis delineates myeloid and lymphoid compartment remodeling during successful Immune-Checkpoint cancer therapy. Cell 2018;175:1443 10.1016/j.cell.2018.11.003 30445041 PMC6541402

[11] Rudd CE A new perspective in cancer immunotherapy: PD-1 on myeloid cells takes center stage in orchestrating immune checkpoint blockade. Science Immunology 2020;5:eaaz8128 10.1126/sciimmunol.aaz8128 31901075

[12] Wang F, Li B, Wei Y, et al Tumor-Derived exosomes induce PD1+ macrophage population in human gastric cancer that promotes disease progression. Oncogenesis 2018;7:41 10.1038/s41389-018-0049-3 29799520 PMC5968036

[13] Gordon SR, Maute RL, Dulken BW, et al Pd-1 expression by tumour-associated macrophages inhibits phagocytosis and tumour immunity. Nature 2017;545:495–9. 10.1038/nature22396 28514441 PMC5931375

[14] Dhupkar P, Gordon N, Stewart J, et al Anti-Pd-1 therapy redirects macrophages from an M2 to an M1 phenotype inducing regression of os lung metastases. Cancer Med 2018;7:2654–64. 10.1002/cam4.1518 29733528 PMC6010882

[15] Strauss L, Mahmoud MAA, Weaver JD Targeted deletion of PD-1 in myeloid cells induces antitumor immunity 2020;5. 10.1126/sciimmunol.aay1863 PMC7183328 31901074

[16] Chen W, Wang J, Jia L, et al Attenuation of the programmed cell death-1 pathway increases the M1 polarization of macrophages induced by zymosan. Cell Death Dis 2016;7:e2115 10.1038/cddis.2016.33 26913605 PMC4849159

[17] Huang X, Venet F, Wang YL, et al Pd-1 expression by macrophages plays a pathologic role in altering microbial clearance and the innate inflammatory response to sepsis. Proc Natl Acad Sci U S A 2009;106:6303–8. 10.1073/pnas.0809422106 19332785 PMC2669369

[18] Tsukamoto H, Fujieda K, Miyashita A, et al Combined blockade of IL6 and PD-1/PD-L1 signaling abrogates mutual regulation of their immunosuppressive effects in the tumor microenvironment. Cancer Res 2018;78:5011–22. 10.1158/0008-5472.CAN-18-0118 29967259

[19] Kaplanov I, Carmi Y Blocking IL-1β reverses the immunosuppression in mouse breast cancer and synergizes with anti-PD-1 for tumor abrogation 2019;116:1361–9. 10.1073/pnas.1812266115 PMC6347724 30545915

[20] Qorraj M, Bruns H, Böttcher M, et al The PD-1/PD-L1 axis contributes to immune metabolic dysfunctions of monocytes in chronic lymphocytic leukemia. Leukemia 2017;31:470–8. 10.1038/leu.2016.214 27479178

[21] Yao A, Liu F, Chen K, et al Programmed death 1 deficiency induces the polarization of macrophages/microglia to the M1 phenotype after spinal cord injury in mice. Neurotherapeutics 2014;11:636–50. 10.1007/s13311-013-0254-x 24853068 PMC4121443

[22] Rashidian M, LaFleur MW, Verschoor VL, et al Immuno-PET identifies the myeloid compartment as a key contributor to the outcome of the antitumor response under PD-1 blockade 2019;116:16971–80. 10.1073/pnas.1905005116 PMC6708368 31375632

[23] Lyford-Pike S, Peng S, Young GD, et al Evidence for a role of the PD-1:PD-L1 pathway in immune resistance of HPV-associated head and neck squamous cell carcinoma. Cancer Res 2013;73:1733–41. 10.1158/0008-5472.CAN-12-2384 23288508 PMC3602406

[24] Tumeh PC, Harview CL, Yearley JH, et al Pd-1 blockade induces responses by inhibiting adaptive immune resistance. Nature 2014;515:568–71. 10.1038/nature13954 25428505 PMC4246418

[25] Xiong H, Mittman S, Rodriguez R, et al Anti–PD-L1 treatment results in functional remodeling of the macrophage compartment. Cancer Res 2019;79:1493–506. 10.1158/0008-5472.CAN-18-3208 30679180

[26] Diskin B, Adam S, Cassini MF, et al Pd-L1 engagement on T cells promotes self-tolerance and suppression of neighboring macrophages and effector T cells in cancer 2020;21:442–54. 10.1038/s41590-020-0620-x 32152508

[27] Nguyen LT, Ohashi PS Clinical blockade of PD1 and LAG3 — potential mechanisms of action. Nat Rev Immunol 2015;15:45–56. 10.1038/nri3790 25534622

[28] Kleinovink JW, Marijt KA, Schoonderwoerd MJA, et al Pd-L1 expression on malignant cells is no prerequisite for checkpoint therapy. Oncoimmunology 2017;6:e1294299 10.1080/2162402X.2017.1294299 28507803 PMC5414865

[29] Taube JM, Klein A, Brahmer JR, et al Association of PD-1, PD-1 ligands, and other features of the tumor immune microenvironment with response to Anti–PD-1 therapy. Clinical Cancer Research 2014;20:5064–74. 10.1158/1078-0432.CCR-13-3271 24714771 PMC4185001

[30] Tang H, Liang Y, Anders RA, et al Pd-L1 on host cells is essential for PD-L1 blockade–mediated tumor regression. Journal of Clinical Investigation 2018;128:580–8. 10.1172/JCI96061 PMC5785245 29337303

[31] Cheng Y, Li H, Deng Y, et al Cancer-Associated fibroblasts induce PDL1+ neutrophils through the IL6-STAT3 pathway that foster immune suppression in hepatocellular carcinoma. Cell Death Dis 2018;9:422 10.1038/s41419-018-0458-4 29556041 PMC5859264

[32] Lau J, Cheung J, Navarro A, et al Tumour and host cell PD-L1 is required to mediate suppression of anti-tumour immunity in mice. Nat Commun 2017;8:14572 10.1038/ncomms14572 28220772 PMC5321797

[33] Hartley GP, Chow L Programmed cell death ligand 1 (PD-L1) signaling regulates macrophage proliferation and activation 2018;6:1260–73. 10.1158/2326-6066.CIR-17-0537 30012633

[34] Chaudhri A, Xiao Y, Klee AN, et al Pd-L1 binds to B7-1 only in cis on the same cell surface. cancer immunology research 2018;6:921–9. 10.1158/2326-6066.CIR-17-0316 PMC7394266 29871885

[35] Burrack AL, Spartz EJ, Raynor JF, et al Combination PD-1 and PD-L1 blockade promotes durable Neoantigen-Specific T cell-mediated immunity in pancreatic ductal adenocarcinoma. Cell Rep 2019;28:2140–55. 10.1016/j.celrep.2019.07.059 31433988 PMC7975822

[36] Frasca L, Nasso M, Spensieri F, et al IFN-γ Arms Human Dendritic Cells to Perform Multiple Effector Functions. J Immunol 2008;180:1471–81. 10.4049/jimmunol.180.3.1471 18209042

[37] He T, Tang C, Xu S, et al Interferon gamma stimulates cellular maturation of dendritic cell line DC2.4 leading to induction of efficient cytotoxic T cell responses and antitumor immunity. Cell Mol Immunol 2007;4:105–11. 17484804

[38] Garris CS, Arlauckas SP, Kohler RH, et al Successful anti-PD-1 cancer immunotherapy requires T Cell-Dendritic cell crosstalk involving the cytokines IFN-γ and IL-12. Immunity 2018;49:1148–61. 10.1016/j.immuni.2018.09.024 30552023 PMC6301092

[39] Lim TS, Chew V, Sieow JL, et al PD-1 expression on dendritic cells suppresses CD8 ^+^ T cell function and antitumor immunity. Oncoimmunology 2016;5:e1085146 10.1080/2162402X.2015.1085146 27141339 PMC4839350

[40] Krempski J, Karyampudi L, Behrens MD, et al Tumor-Infiltrating Programmed Death Receptor-1 ^+^Dendritic Cells Mediate Immune Suppression in Ovarian Cancer. The Journal of Immunology 2011;186:6905–13. 10.4049/jimmunol.1100274 21551365 PMC3110549

[41] Park SJ, Namkoong H, Doh J, et al Negative role of inducible PD-1 on survival of activated dendritic cells. J Leukoc Biol 2014;95:621–9. 10.1189/jlb.0813443 24319287

[42] Yao S, Wang S, Zhu Y, et al Pd-1 on dendritic cells impedes innate immunity against bacterial infection. Blood 2009;113:5811–8. 10.1182/blood-2009-02-203141 19339692 PMC2700320

[43] Karyampudi L, Lamichhane P, Krempski J, et al Pd-1 blunts the function of ovarian Tumor–Infiltrating dendritic cells by inactivating NF-κB. Cancer Res 2016;76:239–50. 10.1158/0008-5472.CAN-15-0748 26567141 PMC4715980

[44] Herbst RS, Soria J-C, Kowanetz M, et al Predictive correlates of response to the anti-PD-L1 antibody MPDL3280A in cancer patients. Nature 2014;515:563–7. 10.1038/nature14011 25428504 PMC4836193

[45] Mayoux M, Roller A, Pulko V, et al Dendritic cells dictate responses to PD-L1 blockade cancer immunotherapy 2020;12. 10.1126/scitranslmed.aav7431 32161104

[46] Sponaas A-M, Moharrami NN, Feyzi E, et al PDL1 expression on plasma and dendritic cells in myeloma bone marrow suggests benefit of targeted anti PD1-PDL1 therapy. PLoS One 2015;10:e0139867 10.1371/journal.pone.0139867 26444869 PMC4596870

[47] Yogev N, Frommer F, Lukas D, et al Dendritic cells ameliorate autoimmunity in the CNS by controlling the homeostasis of PD-1 Receptor+ regulatory T cells. Immunity 2012;37:264–75. 10.1016/j.immuni.2012.05.025 22902234

[48] SA O, D-C W, Cheung J, et al Pd-L1 expression by dendritic cells is a key regulator of T-cell immunity in cancer. Nature Cancer 2020;1:681–91. 10.1038/s43018-020-0075-x 35122038

[49] Peng Q, Qiu X, Zhang Z, et al Pd-L1 on dendritic cells attenuates T cell activation and regulates response to immune checkpoint blockade. Nat Commun 2020;11:4835 10.1038/s41467-020-18570-x 32973173 PMC7518441

[50] Butte MJ, Keir ME, Phamduy TB, et al Programmed death-1 ligand 1 interacts specifically with the B7-1 costimulatory molecule to inhibit T cell responses. Immunity 2007;27:111–22. 10.1016/j.immuni.2007.05.016 17629517 PMC2707944

[51] Radford KJ, Tullett KM, Lahoud MH Dendritic cells and cancer immunotherapy. Curr Opin Immunol 2014;27:26–32. 10.1016/j.coi.2014.01.005 24513968

[52] Gabrilovich DI, Ostrand-Rosenberg S, Bronte V Coordinated regulation of myeloid cells by tumours. Nat Rev Immunol 2012;12:253–68. 10.1038/nri3175 22437938 PMC3587148

[53] Weber J, Gibney G, Kudchadkar R, et al Phase I/II study of metastatic melanoma patients treated with nivolumab who had progressed after ipilimumab. Cancer Immunology Research 2016;4:345–53. 10.1158/2326-6066.CIR-15-0193 26873574 PMC4818672

[54] Theivanthiran B, Evans KS, DeVito NC, et al A tumor-intrinsic PD-L1/NLRP3 inflammasome signaling pathway drives resistance to anti–PD-1 immunotherapy. Journal of Clinical Investigation 2020;130:2570–86. 10.1172/JCI133055 PMC7190922 32017708

[55] Ribas A, Shin DS, Zaretsky J, et al Pd-1 blockade expands intratumoral memory T cells. Cancer Immunology Research 2016;4:194–203. 10.1158/2326-6066.CIR-15-0210 26787823 PMC4775381

[56] Neubert NJ, Schmittnaegel M T cell-induced CSF1 promotes melanoma resistance to PD1 blockade 2018;10. 10.1126/scitranslmed.aan3311 PMC5957531 29643229

[57] Steele CW, Karim SA, Leach JDG, et al Cxcr2 inhibition profoundly suppresses metastases and augments immunotherapy in pancreatic ductal adenocarcinoma. Cancer Cell 2016;29:832–45. 10.1016/j.ccell.2016.04.014 27265504 PMC4912354

[58] Nam S, Lee A, Lim J, et al Analysis of the expression and regulation of PD-1 protein on the surface of myeloid-derived suppressor cells (MDSCs). Biomol Ther 2019;27:63–70. 10.4062/biomolther.2018.201 PMC6319546 30521746

[59] Lu C, Redd PS, Lee JR, et al The expression profiles and regulation of PD-L1 in tumor-induced myeloid-derived suppressor cells. Oncoimmunology 2016;5:e1247135 10.1080/2162402X.2016.1247135 28123883 PMC5214087

[60] Görgün G, Samur MK, Cowens KB, et al Lenalidomide enhances immune checkpoint Blockade-Induced immune response in multiple myeloma. Clinical Cancer Research 2015;21:4607–18. 10.1158/1078-0432.CCR-15-0200 25979485 PMC4609232

[61] GT Y, LL B, Huang CF, et al Pd-1 blockade attenuates immunosuppressive myeloid cells due to inhibition of CD47/SIRPalpha axis in HPV negative head and neck squamous cell carcinoma. Oncotarget 2015;6:42067–80. 26573233 10.18632/oncotarget.5955 PMC4747210

[62] Noman MZ, Desantis G, Janji B, et al Pd-L1 is a novel direct target of HIF-1α, and its blockade under hypoxia enhanced MDSC-mediated T cell activation. Journal of Experimental Medicine 2014;211:781–90. 10.1084/jem.20131916 PMC4010891 24778419

[63] Ballbach M, Dannert A, Singh A, et al Expression of checkpoint molecules on myeloid-derived suppressor cells. Immunol Lett 2017;192:1–6. 10.1016/j.imlet.2017.10.001 28987474

[64] Benson DM, Bakan CE, Mishra A, et al The PD-1/PD-L1 axis modulates the natural killer cell versus multiple myeloma effect: a therapeutic target for CT-011, a novel monoclonal anti–PD-1 antibody. Blood 2010;116:2286–94. 10.1182/blood-2010-02-271874 20460501 PMC3490105

[65] Tumino N, Martini S, Munari E, et al Presence of innate lymphoid cells in pleural effusions of primary and metastatic tumors: functional analysis and expression of PD-1 receptor 2019;145:1660–8. 10.1002/ijc.32262 PMC6767381 30856277

[66] Damele L, Ottonello S, Mingari MC, et al Targeted Therapies: Friends or Foes for Patient’s NK Cell-Mediated Tumor Immune-Surveillance? Cancers 2020;12:774 10.3390/cancers12040774 PMC7226262 32218226

[67] Vivier E, Artis D, Colonna M, et al Innate lymphoid cells: 10 years on. Cell 2018;174:1054–66. 10.1016/j.cell.2018.07.017 30142344

[68] Shifrin N, Raulet DH, Ardolino M Nk cell self tolerance, responsiveness and missing self recognition. Semin Immunol 2014;26:138–44. 10.1016/j.smim.2014.02.007 24629893 PMC3984600

[69] Judge SJ, Dunai C, Aguilar EG, et al Minimal PD-1 expression in mouse and human NK cells under diverse conditions. J Clin Invest 2020;130:3051–68. 10.1172/JCI133353 32134744 PMC7260004

[70] Pesce S, Greppi M, Tabellini G, et al Identification of a subset of human natural killer cells expressing high levels of programmed death 1: A phenotypic and functional characterization. Journal of Allergy and Clinical Immunology 2017;139:335–46. 10.1016/j.jaci.2016.04.025 27372564

[71] Hsu J, Hodgins JJ, Marathe M, et al Contribution of NK cells to immunotherapy mediated by PD-1/PD-L1 blockade. Journal of Clinical Investigation 2018;128:4654–68. 10.1172/JCI99317 PMC6159991 30198904

[72] Wiesmayr S, Webber SA, Macedo C, et al Decreased NKp46 and NKG2D and elevated PD-1 are associated with altered NK-cell function in pediatric transplant patients with PTLD. Eur J Immunol 2012;42:541–50. 10.1002/eji.201141832 22105417 PMC3607363

[73] Dong W, Wu X, Ma S, et al The mechanism of anti-PD-L1 antibody efficacy against PD-L1-Negative tumors identifies NK cells expressing PD-L1 as a cytolytic effector 2019;9:1422–37. 10.1158/2159-8290.CD-18-1259 PMC7253691 31340937

[74] Mariotti FR, Quatrini L, Munari E, et al Innate lymphoid cells: expression of PD-1 and other checkpoints in normal and pathological conditions. Front Immunol 2019;10:910 10.3389/fimmu.2019.00910 31105707 PMC6498986

[75] Yu Y, Tsang JCH, Wang C, et al Single-Cell RNA-seq identifies a PD-1hi ILC progenitor and defines its development pathway. Nature 2016;539:102–6. 10.1038/nature20105 27749818

[76] Moral JA, Leung J, Rojas LA, et al Ilc2S amplify PD-1 blockade by activating tissue-specific cancer immunity. Nature 2020;579:130–5. 10.1038/s41586-020-2015-4 32076273 PMC7060130

[77] Vacca P, Pesce S, Greppi M, et al Pd-1 is expressed by and regulates human group 3 innate lymphoid cells in human decidua. Mucosal Immunol 2019;12:624–31. 10.1038/s41385-019-0141-9 30755717

[78] GT Y, LL B, Zhao YY, et al Ctla4 blockade reduces immature myeloid cells in head and neck squamous cell carcinoma. Oncoimmunology 2016;5:e1151594. 27471622 10.1080/2162402X.2016.1151594 PMC4938362

[79] de Coaña YP, Poschke I, Gentilcore G, et al Ipilimumab treatment results in an early decrease in the frequency of circulating granulocytic myeloid-derived suppressor cells as well as their Arginase1 production. Cancer Immunology Research 2013;1:158–62. 10.1158/2326-6066.CIR-13-0016 24777678

[80] Arce Vargas F, Furness AJS, Litchfield K, et al Fc effector function contributes to the activity of human anti-CTLA-4 antibodies. Cancer Cell 2018;33:649–63. 10.1016/j.ccell.2018.02.010 29576375 PMC5904288

[81] Stojanovic A, Fiegler N, Brunner-Weinzierl M, et al Ctla-4 is expressed by activated mouse NK cells and inhibits NK cell IFN-γ production in response to mature dendritic cells. The Journal of Immunology 2014;192:4184–91. 10.4049/jimmunol.1302091 24688023

[82] Andrews LP, Marciscano AE, Drake CG, et al LAG3 (CD223) as a cancer immunotherapy target. Immunol Rev 2017;276:80–96. 10.1111/imr.12519 28258692 PMC5338468

[83] Workman CJ, Wang Y, El Kasmi KC, et al LAG-3 regulates plasmacytoid dendritic cell homeostasis. The Journal of Immunology 2009;182:1885–91. 10.4049/jimmunol.0800185 19201841 PMC2675170

[84] Keane C, Law SC, Gould C, et al LAG3: a novel immune checkpoint expressed by multiple lymphocyte subsets in diffuse large B-cell lymphoma. Blood Advances 2020;4:1367–77. 10.1182/bloodadvances.2019001390 32267932 PMC7160288

[85] Ma Q, Liu J, Wu G, et al Co-Expression of LAG3 and Tim3 identifies a potent Treg population that suppresses macrophage functions in colorectal cancer patients. Clin Exp Pharmacol Physiol 2018;45:1002–9. 10.1111/1440-1681.12992 29905955

[86] Stanietsky N, Rovis TL, Glasner A, et al Mouse TIGIT inhibits NK-cell cytotoxicity upon interaction with Pvr. European journal of immunology 2013;43:2138–50. 23677581 10.1002/eji.201243072 PMC3863769

[87] Sarhan D, Cichocki F, Zhang B, et al Adaptive NK cells with low TIGIT expression are inherently resistant to myeloid-derived suppressor cells. Cancer Res 2016;76:5696–706. 10.1158/0008-5472.CAN-16-0839 27503932 PMC5050142

[88] Wu L, Mao L, Liu J-F, et al Blockade of TIGIT/CD155 signaling reverses T-cell exhaustion and enhances antitumor capability in head and neck squamous cell carcinoma. Cancer Immunology Research 2019;7:1700–13. 10.1158/2326-6066.CIR-18-0725 31387897

[89] Kurtulus S, Sakuishi K, Ngiow S-F, et al Tigit predominantly regulates the immune response via regulatory T cells. J Clin Invest 2015;125:4053–62. 10.1172/JCI81187 26413872 PMC4639980

[90] Li M, Xia P, Du Y, et al T-Cell immunoglobulin and ITIM domain (TIGIT) receptor/poliovirus receptor (Pvr) ligand engagement suppresses interferon-γ production of natural killer cells via β-arrestin 2-mediated negative signaling. Journal of Biological Chemistry 2014;289:17647–57. 10.1074/jbc.M114.572420 PMC4067199 24817116

[91] Wolf Y, Anderson AC, Kuchroo VK Tim3 comes of age as an inhibitory receptor. Nat Rev Immunol 2020;20:173–85. 10.1038/s41577-019-0224-6 31676858 PMC7327798

[92] Anderson AC Tim-3: an emerging target in the cancer immunotherapy landscape. Cancer Immunology Research 2014;2:393–8. 10.1158/2326-6066.CIR-14-0039 24795351

[93] Sabatos CA, Chakravarti S, Cha E, et al Interaction of Tim-3 and Tim-3 ligand regulates T helper type 1 responses and induction of peripheral tolerance. Nat Immunol 2003;4:1102–10. 10.1038/ni988 14556006

[94] Liu J-F, Ma S-R, Mao L, et al T-Cell immunoglobulin mucin 3 blockade drives an antitumor immune response in head and neck cancer. Mol Oncol 2017;11:235–47. 10.1002/1878-0261.12029 28102051 PMC5527458

[95] Anderson AC, Anderson DE, Bregoli L, et al Promotion of tissue inflammation by the immune receptor Tim-3 expressed on innate immune cells. Science 2007;318:1141–3. 10.1126/science.1148536 18006747

[96] Chiba S, Baghdadi M, Akiba H, et al Tumor-Infiltrating DCs suppress nucleic acid–mediated innate immune responses through interactions between the receptor Tim-3 and the alarmin HMGB1. Nat Immunol 2012;13:832–42. 10.1038/ni.2376 22842346 PMC3622453

[97] Á deMP, Gardner A, Hiebler S, et al TIM-3 Regulates CD103(+) Dendritic Cell Function and Response to Chemotherapy in Breast Cancer. Cancer cell 2018;33:60–74. 29316433 10.1016/j.ccell.2017.11.019 PMC5764109

